# Simultaneous Detection of Omicron and Other SARS-CoV-2 Variants by Multiplex PCR MassARRAY Technology

**DOI:** 10.21203/rs.3.rs-2482226/v1

**Published:** 2023-01-17

**Authors:** Supaporn Wacharapluesadee, Piyapha Hirunpatrawong, Sininat Petcharat, Pattama Torvorapanit, Anusara Jitsatja, Nattakarn Thippamom, Sasiprapa Ninwattana, Chanchanit Phanlop, Rome Buathong, Ratanaporn Tangwangvivat, Chonticha Klungthong, Piyawan Chinnawirotpisan, Taweewun Hunsawong, Krairerk Suthum, Suparerk Komolsiri, Anthony R. Jones, Stefan Fernandez, Opass Putcharoen

**Affiliations:** Thai Red Cross Emerging Infectious Diseases Clinical Center, King Chulalongkorn Memorial Hospital, Bangkok, Thailand; Thai Red Cross Emerging Infectious Diseases Clinical Center, King Chulalongkorn Memorial Hospital, Bangkok, Thailand; Thai Red Cross Emerging Infectious Diseases Clinical Center, King Chulalongkorn Memorial Hospital, Bangkok, Thailand; Thai Red Cross Emerging Infectious Diseases Clinical Center, King Chulalongkorn Memorial Hospital, Bangkok, Thailand; Thai Red Cross Emerging Infectious Diseases Clinical Center, King Chulalongkorn Memorial Hospital, Bangkok, Thailand; Thai Red Cross Emerging Infectious Diseases Clinical Center, King Chulalongkorn Memorial Hospital, Bangkok, Thailand; Thai Red Cross Emerging Infectious Diseases Clinical Center, King Chulalongkorn Memorial Hospital, Bangkok, Thailand; Thai Red Cross Emerging Infectious Diseases Clinical Center, King Chulalongkorn Memorial Hospital, Bangkok, Thailand; Division of International Communicable Disease Control Ports and Quarantine, Department of Diseases Control, Ministry of Public Health, Nonthaburi, Thailand; Division of Communicable Diseases, Department of Diseases Control, Ministry of Public Health, Nonthaburi, Thailand; Department of Virology, Armed Forces Research Institute of Medical Sciences, Bangkok, Thailand; Department of Virology, Armed Forces Research Institute of Medical Sciences, Bangkok, Thailand; Department of Virology, Armed Forces Research Institute of Medical Sciences, Bangkok, Thailand; Office of Disease Prevention and Control, Region 5, Department of Diseases Control, Ministry of Public Health, Ratchaburi, Thailand; Office of Disease Prevention and Control, Region 5, Department of Diseases Control, Ministry of Public Health, Ratchaburi, Thailand; Department of Virology, Armed Forces Research Institute of Medical Sciences, Bangkok, Thailand; Department of Virology, Armed Forces Research Institute of Medical Sciences, Bangkok, Thailand; Thai Red Cross Emerging Infectious Diseases Clinical Center, King Chulalongkorn Memorial Hospital, Division of Infectious Diseases, Department of Medicine, Faculty of Medicine, Chulalongkorn University, Bangkok, Thailand

**Keywords:** SARS-CoV-2 variants, MALDI-TOF MS, MassARRAY, Multiplex PCR, Variants of Concern (VOCs)

## Abstract

The rapid emergence of SARS-CoV-2 variants with high severity and transmutability adds further urgency for rapid and multiplex molecular testing to identify the variants. A nucleotide matrix-assisted laser-desorption-ionization time-of-flight mass spectrophotometry (MALDI-TOF MS)-based assay was developed (called point mutation array, PMA) to identify four major SARS-CoV-2 variants of concern (VOCs) including Alpha, Beta, Delta, and Omicron (namely PMA-ABDO) and differentiate Omicron subvariant (namely PMA-Omicron). PMA-ABDO and PMA-Omicron consist of 24 and 28 mutation sites of the spike gene. Both PMA panels specifically identified VOCs with as low as 10 viral copies/ μl. The panel has shown a 100% concordant with the Next Generation Sequencing (NGS) results testing on 256 clinical specimens with real-time PCR cycle threshold (Ct) values less than 26. It showed a higher sensitivity over NGS; 25/28 samples were positive by PMA but not NGS in the clinical samples with PCR Ct higher than 26. Due to the mass of nucleotide used to differentiate between wild-type and mutation strains, the co-infection or recombination of multiple variants can be determined by the PMA method. This method is flexible in adding a new primer set to identify a new emerging mutation site among the current circulating VOCs and the turnaround time is less than 8 hours. However, the spike gene sequencing or NGS retains the advantage of detecting newly emerged variants.

## Introduction

COVID-19 is an infectious disease caused by severe acute respiratory syndrome coronavirus 2 (SARS-CoV-2) and has spread worldwide since early 2020. To date, five SARS-CoV-2 variants of concern (VOCs) have been identified by World Health Organization (WHO), including Alpha (B.1.1.7, firstly identified in the UK in Sep-2020), Beta (B.1.351, firstly identified in South Africa in May-2020), Gamma (P.1, firstly reported in Brazil in Nov-2020), Delta (B.1.617.2, first recognized in India in Oct-2020) and Omicron (B.1.529, first notified to WHO from South Africa on Nov-2021) [1]. Currently, the Omicron variant is the dominant variant worldwide. Omicron is a highly mutated SARS-CoV-2 variant. As of 30 April 2022, 3,147,505 sequences with the Omicron combined lineage with at least 74 sub-lineage such as BA.1, BA.1.1, BA.2, and BA.3 have been detected worldwide since the combined lineage was identified [2]. SARS-CoV-2 Omicron has 39 mutations in the Spike (S) gene, of which 27 sites were new [3]. Omicron has more than twice the number of mutation sites compared to Delta, mainly in the spike/ACE2 interaction region. The insertion of ins214E, ins215P, and ins216E was detected for the first time in the Omicron S gene among the 11 SARS-CoV-2 variants. This raises the concerns that it may contribute to the high transmissibility of Omicron and highlights the need to identify this variant rapidly for efficient prevention and control.

Whole-genome sequencing (WGS) or targeted sequencing of the spike gene using next-generation sequencing (NGS) or Sanger sequencing are the gold standards for identifying and confirming SARS-CoV-2 variants [4]. While highly sensitive and specific, these techniques are time-consuming, costly, require extensive data processing, and are challenging to perform in real-time. Quantitative PCR-based variant analysis of SARS-CoV-2 is rapid, economical, and a potential alternative method to screen for variants [5-6]. However, the detection of this approach is limited to three to four targets of the mutation site in one reaction, which may lead to misidentification when the new variant emerges. For example, three targets of DEL69/70, E484K, and N501Y included in one real-time PCR commercial kit are not sufficient to distinguish the Omicron from the Alpha variant [6]. Due to the emergence of new SARS-CoV-2 variants, there is an urgent need for a multisite detection system that is adaptable, rapid, and accurate for SARS-CoV-2 variant identification. Such a system should be applicable for the early screening and detection of both currently circulating and novel variants.

**Figure 1:**
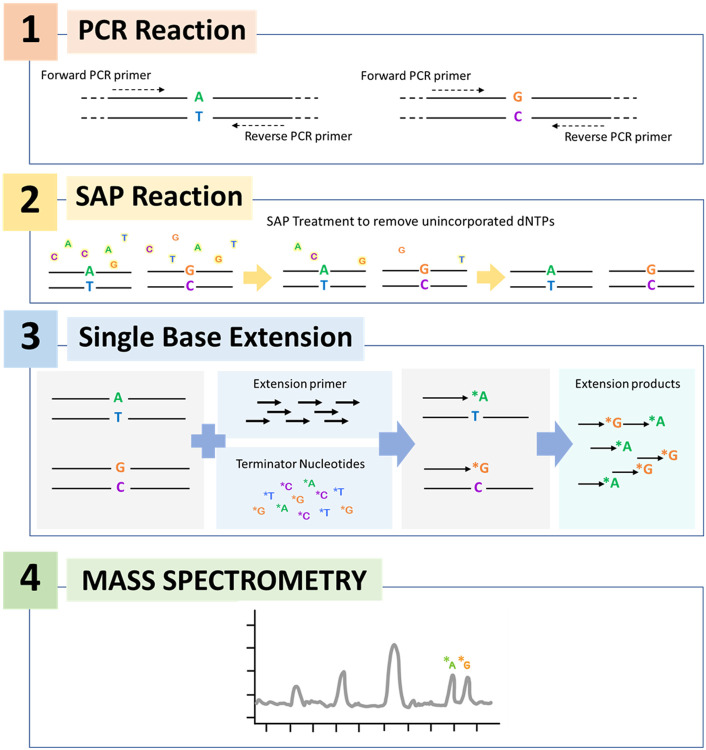
The molecular principle of the MassARRAY method. The analysis begins with reverse transcription of viral RNA into cDNA and amplification by multiplex PCR in a single reaction. Next, PCR products are treated with Shrimp Alkaline Phosphatase (SAP) enzyme that removes unincorporated nucleotides, and a multiplex primer extension (MPE) reaction is carried out. In the MPE reaction, each product is detected at a specific mass because of the addition of terminating nucleotides, which are complementary to the template sequence.

MassARRAY technology is a molecular technique deploying a nucleotide matrix-assisted laser desorption ionization time-of-flight mass spectrometry (MALDI-TOF MS) which has been utilized in the clinical laboratory setting for detecting single nucleotide polymorphism of 50 types of four cancer-driving mutations, as well as detection of *Mycobacterium tuberculosis* and resistance mutations of eight antibiotics [7-8]. The extension primer is specifically designed to bind to the target at the position of one base before the mutation site. MALDI-TOF MS identifies the mass of the extended primer, which is subsequently used to differentiate the mutation type (Fig. 1). The mass primer extension used in the detection step can be designed to detect either gene substitution, insertion, or deletion. Recently, the commercial kit for the qualitative detection of SARS-CoV-2 using RT-PCR reaction together with the MassARRAY system (Agena Bioscience^®^, San Diego, CA, USA) has been developed, and it showed superiority over Real-time PCR assay [R^9^]. This assay amplified five targets (two regions of the ORF gene and three regions of the N gene) simultaneously but did not include the spike gene. It was able to discriminate mutation within the viral genome but could not identify the variants of SARS-CoV-2.

Recently, a novel strategy for detecting SARS-CoV-2 variants using multiplex PCR-mass spectrometry minisequencing technique has been developed [10]. This method is based on the single-base mass probe extension of multiplex PCR amplification products and detected by MALDI-TOF-MS. It was established to detect nine mutation sites (DEL69/70, N501Y, K417N, P681H, D614G, E484K, L452R, E484Q, and P681R) in the receptor-binding domain of the spike protein of SARS-CoV-2 variants in one reaction, enabling the detection of three VOCs including Alpha, Beta, and Delta variants but not the Omicron variant.

This study extended the MassARRY technology to simultaneously detect and identify multiple mutation sites of the SARS-CoV-2 spike gene. Two panels of point mutations array (PMA) were developed (1) PMA-ABDO for screening 24 mutation sites for four VOCs, including Alpha, Beta, Delta, and Omicron, which facilitates (2) PMA-Omicron for specific detection of 28 mutation sites for Omicron confirmation. The sensitivity and specificity of the test were validated using virus isolates and 40 non-COVID-19 clinical samples, respectively. Finally, 284 clinical samples with known SARS-CoV-2 lineages identified by the NGS method were used to compare the specificity of PMA and NGS.

## Results

### 1. Performance of PMA

**Figure 2:**
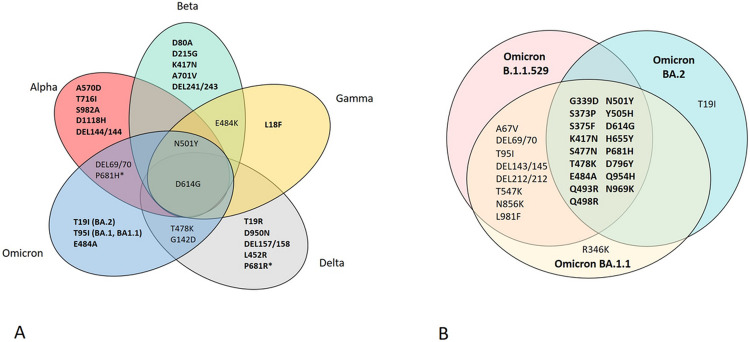
**2A.** Mutation sites at the spike gene used in PMA-ABDO. Twenty-four mutation sites were selected from the prevalence across the lineage; the bold text is the hallmark mutation set for each variant. *P681H and P681R sites were amplified and detected with the same primer set, differentiated by A and G nucleotide mass, respectively. **2B.** Mutation sites at the spike gene used in PMA-Omicron to detect B.1.1529, BA.1.1. and BA.2 sublineage of Omicron. Twenty-eight mutation sites were selected from the prevalence across the lineage; 27 mutation sites (showed in this figure) were specific to Omicron lineage, and 17 (bold text) were found on three sublineages of Omicron. D1118H (not shown in the figure) is the specific mutation target for the Alpha variant; it was included in the PMA-Omicron assay as a control.

The mutation sites at the spike gene were chosen as a detection target to characterize the SARS-CoV-2 VOCs in this study. Table 1 shows a list of the mutation site and prevalence across lineages used in this study. It should be noted that L18F was included to differentiate the Gamma variant as its prevalence was 97.9%. The SARS-CoV-2 nucleoprotein (N) gene target was included in the PMA panels as the internal control to validate the assay sensitivity and performance. The PMA-ABDO panel consists of 24 specific mutation sites of the spike gene for detecting 4 VOCs (Alpha, Beta, Delta, and Omicron) simultaneously in one reaction (Fig. 2A). The total gene target was 25 (Table 1), consisting of 25 extension primers with 22 forward and 21 reverse primers (available upon request). The second panel, the PMA Omicron panel, consists of 28 spike gene targets in which 20 targets are uniquely specific to Omicron. It can differentiate BA.2 from BA.1 and BA.1.1 variants in one reaction (Fig. 2B). The total gene target for PMA-Omicron is 29 (Table 2), consisting of 29 extension primers with 27 forward and 27 reverse primers (available upon request).

The PMA cutoff was calculated from the mean % call rate of five viral isolates at 5 dilutions (10–10^6^ copies/μl) using GraphPad Prism 9.0 version (Supplement Table 1). It was determined as 43.59%, with 100% sensitivity (95% confidence interval (CI) [86.68%, 100%]) and 100% specificity (95% CI [83.89%, 100.0%]). In this study, the PMA validation criteria consist of (1) positive on the N gene and (2) the total % call rate higher than the cutoff, 43.59%.

### 2. Specificity of the PMA method

Five viral isolates (1,000 copies/ μl) of SARS-CoV-2 ancestral, Alpha, Beta, Delta, and Omicron strains were used to evaluate the specificity of PMA-ABDO and PMA-Omicron assays. All mutation sites were identified correctly in all viral isolates for PMA-ABDO and PMA-Omicron panels (Supplement Tables 2 and 3).

Twenty respiratory samples collected from non-COVID-19 patients who tested negative for SARS-COV-2 RT-PCR and other common respiratory viruses were used to evaluate non-specific signal of the PMA-ABDO assay. No call rate was detected on all 24 mutation sites and N gene in all samples; only unextended primer (UEP) signals were found (Supplement Table 4).

A total of twenty nucleic acid samples tested positive for other respiratory pathogens, including adenovirus (1), influenza (Flu) A/H1N1 2009 (2), Flu A/H3 (2), Flu B (2), enterovirus 71 (1), coxsackievirus A6 (1), bocavirus (1), human coronavirus (HCoV)-229E (1), HCoV–OC43 (2), HCoV-NL63 (1), HCoV-HKU1 (1), parainfluenza virus (3), metapneumovirus (1), and respiratory syncytial (1) were used for evaluating the cross-reactivity of the PMA primers. N gene was not detected among all the tested samples. Non-specific signals were found in 4 gene targets; D80A (n = 13), T95I (n = 2), A570D (n = 5), and D614G (n = 2) (Supplement Table 4). The call rate range was 0–8% (1–2 non-specific peaks were found from 24 mutation sites). According to our justification criteria, these specimens were interpreted as SARS-CoV-2 negative and require no further analysis for variant identification. The overall specificity determined from non-COVID-19 samples (n = 40) of PMA-ABDO was 100% (95% CI [86.68%, 100.0%]) (Supplement Table 4).

### 3. Sensitivity of the PMA methods

Five ten-fold serial dilutions of viral RNA (10 to 10^6^ copies/μl) from five SARS-CoV-2 isolates of ancestral, Alpha, Beta, Delta, and Omicron strains, were used to evaluate the sensitivity on each mutation site of PMA-ABDO and PMA-Omicron panels. The detection limit of each mutation site varied between 10 to 1,000 copies/ μl; overall sensitivity was 10 copies/ μl. The detection limit of the PMA-ABDO was 10 copies/μl on 19, 20, 21, 20, and 20 mutation sites, 100 copies/μl on 4, 3, 1, 2, and 3 mutation sites, and 1,000 copies/μl on 1, 1, 2, 2, and 1 mutation sites, for ancestral, Alpha, Beta, Delta, and Omicron viral strains respectively (Supplement Table 2). The detection limit of the PMA-Omicron was 10 copies/μl on 22, 27, 26, 23, and 27 mutation sites, 100 copies/μl on 4, 1, 1, 3, and 1 mutation sites, and 1,000 copies/μl on 2, 0, 1, 2, and 0 mutation sites for ancestral, Alpha, Beta, Delta, and Omicron viral strains, respectively (Supplement Table 3).

### 4. Sensitivity and specificity comparisons between PMA and NGS methods

WGS is considered the gold standard for SARS-CoV-2 variant identification. A total of 256 SARS-CoV-2 positive samples (PCR cycle threshold (Ct) values of N gene less than 26) were analyzed using PMA-ABDO or PMA-Omicron, and the results were compared with those obtained by the NGS method (Group 1, Table 3). We found that the variant identification by PMA (call rate > 43.59%) was concordant with the NGS results. Out of 197 samples, both NGS and PMA-ABDO methods could detect 174 samples of VOCs, including 58 of Alpha, 3 of Beta, 112 of Delta, and 1 of Gamma, and 23 non-VOC samples. Fifty-nine samples were analyzed using PMA-Omicron. There were 20 of B.1.1529/BA.X (identified as BA.1 by NGS), 16 of BA.1.1, and 23 of BA.2 (Group 1, Table 3). It should be noted that Omicron subvariant (B.1.1.529 and BA.1) cannot be differentiated using this PMA-Omicron assay.

Twenty-eight SARS-CoV-2 positive samples (PCR Ct > 26) failed to be detected by NGS. However, the PMA-ABDO was able to identify 11 Alpha and 9 Delta variants, and the PMA-Omicron successfully detected Omicron (B.1.1.529 or BA.x) in 5 samples (Group 2, Table 3). Three samples that failed to be detected by both methods were those with PCR Ct higher than 35. They contained at two unique mutation points (A570D and T716I), permitting them to be characterized as suspected Alpha variant (Group 2, Table 3).

Twenty-three samples were identified as non-VOCs lineage by NGS, including one of A.6, B.1.1.1, B.1.1.10, B.1.158, B.1.275, B.1.466.2, B.1.5, B.1.40, B.6, and 14 of B.1.36.16. Similarly, these samples were identified as non-VOC SARS-CoV-2 variants by PMA-ABDO (Group 1, Table 3). N gene and wildtype nucleotides of 24 mutation sites of the spike gene were detected in all samples by PMA-ABDO panel.

The detection sensitivity and specificity of PMA-ABDO (174/174 samples) and PMA Omicron (59/59 samples) compared to the NGS method were 100% on the samples with PCR Ct < 26. While 25 of 28 (89.28%) samples with PCR Ct > 26 showed positive results only by the PMA method. The overall detection specificity was 100% based on 23 non-VOC samples (Group 1, Table 3) and 40 non-COVID-19 samples (Supplement Table 4).

### 5. Variant typing in clinical samples with a low viral copy (PCR Ct > 30) by PMA methods

Clinical samples with a low amount of viral RNA (RT-PCR Ct ≥ 30), which is hard to characterize SARS-CoV-2 variants by the NGS method, were tested for variant typing using PMA methods. Firstly, the specimens were screened to identify four significant VOCs (Alpha, Beta, Delta, and Omicron) using the PMA-ABDO assay. The possible positive Omicron samples (containing T95I and/or E484A) were further characterized to determine the sub-lineage of the Omicron variant by PMA-Omicron assay. In 74 tested samples, 63 were positive for PMA (call rate > 43.59%), with 41, 14, and 19 were Alpha, Delta, and Omicron (B.1.1.529/BA.X), respectively (Group 3, Table 2).

### 6. Detection of heterozygous spectra

**Figure 3:**
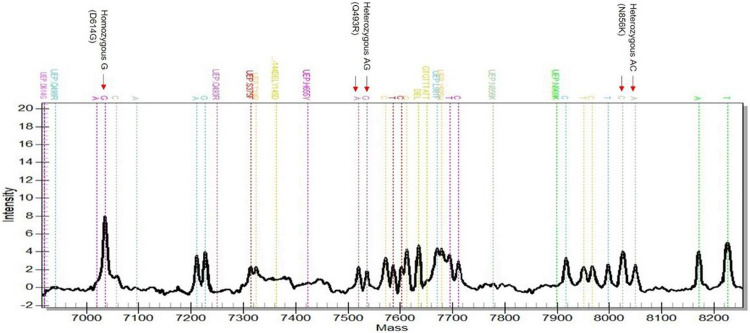
Mass spectra of mock clinical specimens (Delta and Omicron variants) tested by MassARRAY using PMA-Omicron panel. D614G showed a single peak of nucleotide G while heterozygous peaks of wildtype (Delta) and mutation (Omicron) were found at Q493R (nucleotide A and G, respectively) and N856K (nucleotide C and A, respectively) mutation points.

A mixed of Delta and Omicron mock clinical sample was prepared and used to test the ability of MassARRAY technology to distinguish multiple-strains infections. PMA-Omicron was used for the evaluation, and it was found that the D614G site appeared as a single peak since both Delta and Omicron variants have the same nucleotide mutation at this site. The heterozygous spectra were detected and interpreted as co-infection between Delta and Omicron at Q493R (A and G, respectively) and N856K (C and A, respectively) mutation sites (Fig. 3).

## Discussion

Global genomic surveillance for real-time identification of the SARS-CoV-2 variant is a critical public health concern [11]. Whole-genome or complete spike gene sequencing is the gold standard for identifying the strain of SARS-CoV-2. Rapid detection methods with simultaneous detection of variants using real-time multiplex PCR are commercially available and widely used to screen SARS-CoV-2 variants before sequencing confirmation by NGS [12-14]. In a recent study, a similar technology (Multiplex PCR-Mass spectrometry Minisequencing) was developed for the differentiation of three SARS-CoV-2 VOCs, including Alpha, Beta, and Delta, using 9 mutation sites of the spike gene [10]. The detection limit of all 9 sites was 1.5x10^3^ copies. In the other study, nucleic acid mass spectrometry has been previously developed for the simultaneous detection of seven human coronaviruses using 20 viral targets for differentiation; it showed the same sensitivity as that of Real-time PCR assay [15]. However, this method is difficult to identify the new human CoV or SARS-CoV-2 variant because the gene fragments with conserved intraspecies specificity were selected for amplification and detection.

In our study, the MassARRAY technology to detect SARS-CoV-2 VOCs was expanded from the previous study [10] to include the Omicron variant and increase the number of targets to increase the specificity of the assay. Two panels of PMA assay based on the MassARRAY technology were developed to simultaneously detect 24 mutation sites of the SARS-CoV-2 spike gene to identify four VOCs, including Alpha, Beta, Delta, and Omicron, called PMA-ABDO (Fig. 2A). At the same time, a second assay called PMA-Omicron was developed to distinguish Omicron BA.2 from Omicron B.1.1.529 and BA.1 (Fig. 2B). The virus isolates were used to determine the limit of detection and specificity. The overall detection limit of PMA-ABDO against ancestral strain was 10 copies/μl except at P681R/H, where the detection limit was 1,000 copies/μl. The detection limit of MassARRAY technology is affected by both multiplex PCR amplification and multiplex primer extension reactions [10]. Another study has shown that the binding efficiency of the extension primer was reduced when the mutation sites were located in the same PCR fragment, for example, an increase in a detection limit of P681R/H to 1,560 copies compared to 400 copies of D614G [10]. A similar result was found in our study, the detection limit of P681R/H tested with five virus isolates; ancestral, Alpha, Beta, Delta, and Omicron strains, was 1,000 copies/μl, while the detection limit of D614G and A701V were 10 copies/μl. This indicates that the binding efficiency of the P681R/H primer is likely to be lower than other mutation sites in the panel. The P681R/H mutation site was found in three SARS-CoV-2 variants, Alpha, Delta, and Omicron; its low sensitivity has a more negligible effect on the overall test performance.

Two hundred fifty-six clinical samples of SARS-CoV-2 were used to compare the detection specificity between PMA and NGS. The detection limit of the NGS assay in our study was PCR Ct less than 26. The detection specificity of PMA-ABDO and PMA-Omicron of known variants and non-variant SARS-CoV-2 was 100% comparable to NGS results (Group 1, Table 3). Additionally, another study on multiplex PCR mass spectrometry has reported 100% accuracy in all samples with PCR Ct less than 27 [10]. When testing 102 unknown SARS-CoV-2 variant clinical samples (PCR Ct 26–40) with PMA-ABDO and PMA-Omicron assays, 99 (97.05%) were tested positive (Group 2 and 3, Table 3). 25 of 28 samples (89.28%) were PMA positive while all were negative by NGS, indicating a higher sensitivity of PMA over NGS in the low viral copy samples. However, three samples with PCR Ct > 35 were tested positive for the SARS-CoV-2 N gene by PMA, but the variant types could not be identified due to the % call rate lower than the PMA cutoff (43.59%).

The non-specific binding of extension primer was not detected among Alpha, Beta, Delta, and Omicron variants and ancestral strains in all spike mutation sites included in PMA-ABDO (24 sites) and PMA-Omicron (28 sites) when tested with known virus isolates at 1,000 copies/μl (Supplement Tables 2 and 3). Moreover, forty nucleic acids from clinical samples, including twenty of various non-SARS-CoV-2 respiratory viruses and 20 non-COVID-19 patients (Supplement Table 4), were correctly identified by PMA-ABDO as non-COVID-19 according to our justification criteria. However, the non-specific peak (1–2 peaks per sample) was detected in the patient samples infected with other respiratory viruses (specimens were collected before 2019), but it was not found in the non-COVID-19 patients collected in 2021. This reveals that the non-specific detection was unlikely from host DNA binding. There are two possibilities for the cross-reactivity; firstly, the high number of primers in 25-plex PCR of PMA-ABDO may cause non-specific binding with non-targeted genes in other viruses. Secondly, other respiratory viruses, such as HCoV-229E, may contain parts of the genome similar to the targeted mutation sites where the primer can bind non-specifically. A recent study has shown that the Omicron variant harbors a unique insertion mutation of HCoV-229E [16]. The insertion mutation may be acquired through template switching involving the genome of HCoV-229E and SARS-CoV-2 in coinfected patients. Further study with more sample numbers is needed to confirm this observation.

The limitation of variant screening tests, either by real-time PCR or multiplex PCR-Mass Spectrometry-based methods, is the ability to detect a new variant [10, 12, 14]. Due to circulating of multiple SARS-CoV-2 variants and rapid mutation, the multiple mutation sites should be included in the same assay or workflow to avoid misidentification. In the PMA-ABDO, the multiple lineage-defining markers were included for the identification of Alpha (A570D, T716I, S982A, D1118H, DEL144/144), Beta (D80A, D215G, K417N, A701V, and DEL241/243), Delta (D950N, DEL157/158, L452R, P681R), and Omicron (T95I, E484A) (Fig. 2A). Moreover, target detection covering current VOCs in our PMA assay could detect the unusual recombinant variants such as Delta-Omicron or the XJ lineage (BA.1 and BA.2) or co-infection [17-18]. The mocked sample, a mixture of Delta and Omicron variants, was tested with PMA-Omicron assay, and the heterozygous spectra (Fig. 3) demonstrated the advantage of using the PMA system for distinguishing co-infections.

The PMA-Omicron used in this study contained the lineage-defining markers (T19I, DEL69/70, A67V, and Q493R) that can differentiate Omicron BA.3 and BA.4/BA.5. However, additional markers on ORF1a (DEL141/1433) or M gene (D3N) is needed to distinguish between BA.4 and BA.5 [2]. In conclusion, the PMA method is a simple screening assay for SARS-CoV-2 variants with high sensitivity and specificity and has a high-throughput format (a maximum of 384 samples can be detected within 8 hours). The PMA assay can detect mixed nucleotides at a single site (heterozygous) and show a promising tool for detecting SARS-CoV-2 recombination or co-infection associated with recognized VOC. The system is flexible, allowing primer sets to be adjustable with the maximum multiplex MPE up to 40 targets, which can help screen SARS-CoV-2 variants at a low cost.

## Methods

### 1. Clinical specimens and virus isolates

A total of 358 SARS-CoV-2 positive (Table 3) and 40 SARS-CoV-2 negative (Supplement Table 4) nasopharyngeal and throat (NT) swabs in viral transport media were used in this study. Detection of SARS-CoV-2 was determined by Allplex^™^ SARS-CoV-2 Real-time PCR Assay (Seegene Inc., Seoul, South Korea) of specific conserved fragments within *RdRp, N,* and *E* viral genes. The real-time PCR was conducted on Bio-Rad CSF96^™^ system according to the manufacturer’s recommendation. Briefly, 17 μL of the One-step RT-PCR Mastermix was added into PCR tubes, and 8 μL of each sample’s nucleic acids, 2019-nCoV positive control, and negative control (RNase-free Water) was added separately into the tubes. The amplification targets of *RdRp, N,* and *E* viral genes were detected through Cal Red 610, Quasar 670, and FAM channels, respectively. Whereas the internal control was detected with HEX channel. The cutoff PCR Ct was ≤ 40 and limit of detection was 100 RNA Copies/reaction. The PCR ct of N gene (less than 26) was used to determine sensitivities and the comparison with NGS.

This study was approved by the Institutional Review Board of the Faculty of Medicine, Chulalongkorn University (Approval IRB No. 361/59, 400/63), and all methods were performed in accordance with the relevant guidelines and regulations. All patients with suspected COVID-19 or acute respiratory infections were enrolled with written informed consent from December 2018 to March 2022. Five virus isolates, including ancestral, Alpha, Beta, Delta, and Omicron strains, were obtained from the Department of Virology, Armed Forces Research Institute of Medical Sciences (AFRIMS) Bangkok, Thailand. SARS-CoV-2 viruses, ancestral lineage (isolate Hong Kong/VM20001061/2020, NR-52282, GISAID accession ID: EPI_ISL_412028), Alpha variant (isolate hCoV-19/England/204820464/20220/NR-54000/ GISAID accession ID: EPI_ISL_683466), Bata variant (isolate hCoV-19/South Africa/KRISP-EC-K005321/2020, NR-54008/GISAID accession ID: EPI_ISL_678570) were obtained through BEI Resources (NIAID, USA). The Delta B.1.617.2 (hCoV-19/Thailand/CU-A21287-NT/2021/ GISAID accession ID: EPI_ISL_2510689) and Omicron GRA (B.1.1.529 + BA.*) (hCoV-19/Thailand/CU-A211051-NPS/2022/ GISAID accession ID: EPI_ISL_14175998) variants were isolated from clinical specimens collected at King Chulalongkorn Memorial Hospital. All isolates were propagated in Vero E6 cells and quantitated by real-time PCR. All isolates were sequenced to characterize SARS-CoV-2 variants by Next Generation Sequencing (NGS). Viral RNA was extracted using the MagPurix^®^ Viral RNA Extraction Kit (Zinexts, Taiwan) or QIAamp Viral RNA kit (Qiagen, Hilden, Germany), according to the manufacturing protocol.

### 2. Primer Design

Each PCR primer set consisted of three constituent primers, including two primers of first-round PCR (forward and reverse primer) and an extension primer for the detecting step (Fig. 1). The mutation sites were selected from variants reported on the mutation database outbreak.info platform [2] and the primers were designed from the reference SARS-CoV-2 genome (NC_045512.2) using the manufacturer’s assisted software (ASSAY DESIGN SUITE Version 2.2, Agena Bioscience, CA, USA). The primer sequences used in this study are available upon request. The PMA-ABDO panel consisted of twenty-five PCR targets; 24 mutation sites in the spike gene targeted to detect Alpha, Beta, Delta, and Omicron variants (Fig. 2A) and 1 N gene. The PMA-Omicron panel consisted of 20 sets of primers unique to the Omicron variant, 8 targets overlapping between Omicron and other VOCs, and 1 N gene control SAR-CoV-2. N gene was included in both of the PMA panels to use as a control. The sample that showed a signal on the spike gene in an absence of a peak of the N gene would be interpreted as SARS-CoV-2 negative.

### 3. Four main steps for the detection of SARS-CoV-2 variants by PMA with MassARRAY technology

#### 3.1 cDNA synthesis and amplification of multiplex PCR.

cDNA was synthesized immediately after RNA extraction with random hexamers using Superscript III reverse transcription kit (Invitrogen, Thermo Fisher Scientific, MA, USA). Multiplex PCR was conducted using iPLEX Pro Assay (Agena Bioscience, CA, USA). The multiplex PCR consisted of 3 μl of PCR master mix reagents (100 nM of forward and reverse primers, 2 nM of MgCl_2_ solution, 500 μM dNTP, 1x PCR buffer, 0.2 unit/μl of PCR enzyme), and 3 μl of cDNA, giving a total volume of 6 μl. The PCR condition involved a pre-denaturation step at 95°C for 2 min, 45 fs at 95°C for 30 sec, 56°C for 30 sec, 72°C for 1 min, and a final extension at 72°C for 5 min.

#### 3.2 Shrimp Alkaline Phosphatase (SAP) Treatment.

The unincorporated dNTPs in the multiplex PCR reaction were removed by SAP. In brief, 2 μl of SAP enzyme mixture (iPLEX^®^ Pro, Agena Bioscience, CA, USA) was added to 6 μl PCR product and incubated at 37°C for 40 min. The reactions were stopped by inactivation at 85°C for 5 min.

#### 3.3 Multiplex primer extension (MPE) reactions.

Selected mutations on the spike gene (24 and 28 targets for PMA-ABDO and PMA-Omicron assays, respectively) and one N gene were analyzed in a single reaction. The MPE reaction was performed with terminator nucleotides (ddNTPs) and the designed extension primers were used to distinguish the mass difference of the mutant nucleotide from the wildtype (Fig. 1). The MPE reaction mixture (ddNTP mix, iPLEX^®^ Buffer Plus, iPLEX^®^ Enzyme, and mix mass extension primers) was added to the SAP treatment product, according to the manufacturer protocol. The extension reactions were carried out at 95°C for 30 seconds and then 95°C for 5 seconds, followed by 5 cycles at 52°C for 5 seconds and 80°C for 5 seconds, for a total of 40 cycles, and then 72°C for 3 minutes. The final product was desalinated using a resin column.

#### 3.4 MassARRAY data acquiring and analysis.

Purified MPE products were transferred to a 96-well spectroCHIP of MassARRAY (Agena Bioscience, CA, USA) using MALDI-TOF mass spectrometry. Genotype Calling was performed in a real-time manner with MassARRAY RT software version 4.1 and analyzed with MassARRAY Typer software.

### 4. Determine cutoff, limit of detection and specificity

The known virus isolates, including ancestral, Alpha, Beta, Delta, and Omicron strains, were obtained from the Department of Virology, AFRIMS, Bangkok, and used to assess the cutoff, limit of detection (LOD), and specificity of the mutation panels. Ten-fold serial dilution of 10 to 10^6^ copies/μL was used to determine the cutoff and LOD of the PMA assays, while the specificity was determined at a concentration of 1,000 copies/ μL. Each RNA sample was tested in triplicate. The % call rate was calculated from the number of positive peaks divided by the total number of expected peaks and multiplied by 100.

The % call rate cutoff was calculated from the correlation between the amount of virus from 10–10^6^ copies/μl and the mean of % call rate from five viral isolates determined by MassARRAY, GraphPad Prism 9.0 was used for cutoff analysis (Supplement Table 1).

### 5. Whole-genome sequencing by Next Generation Sequencing (NGS) and sequence data analysis

The ARTIC protocol v3 and v4 primers were used for viral RNA amplification [19]. DNA library preparation was performed using an Illumina^®^ DNA Prep kit (Illumina, CA, USA), Illumina^®^ RNA Prep with Enrichment and Respiratory Virus Oligos Panel v2, or a Native barcoding Expansion 1–12 (PCR-free) (Oxford Nanopore Technologies, Oxford, UK). Sequencing was achieved using a MiSeq reagent kit v2 (2 x 250 nucleotides) run on an Illumina MiSeq platform (Illumina, CA, USA) or using a MinION Flow Cell (R9.4.1) performed on a MinION platform (Oxford Nanopore Technologies, Oxford, UK). Sequencing data analysis methods were previously described [20-22]. These included the Burrows-Wheeler Aligner (BWA) MEM algorithm [23], which was used for sequence mapping with the Wuhan-Hu-1 genome (GenBank accession number NC_045512.2), and iVar v1.2.2 [24] and SAMtools [25] which were used for primer region trimming and variant calling (Q scores of ≥ 25), respectively. iVar v1.2.2 was also used for the generation of consensus sequences (Q scores of ≥ 25 and depth of coverage [DOC] of ≥ 10×). Ambiguous bases, deletions, and gaps were identified and confirmed by genome-guided assembly with the reference sequences using Trinity v2.8.5 [26]. SARS-CoV-2 lineages and variants were determined using Pangolin v3.1.20 with lineages version 2022-02-28 [27], GISAID clade nomenclature [28] and Nextclade v1.14.0 [29].

### 6. Statistic Analysis

GraphPad Prism 9.0 version Software (GraphPad Software; San Diego, CA, USA) was used for the analysis cutoff and specificity. All results with p-values < 0.05 were considered statistically significant.

## Figures and Tables

**Table 1:** Mutation prevalence across lineages of SARS-CoV-2 variant target sites used in PMA-ABDO panel

Mutation site Amino acid	Mutation sites nucleotides	Mutation prevalence among lineage (%)^#^					
		Alpha	Beta	Delta	Omicron (B.1.529)	BA.1.1	BA.2
L18F^$^	C21614T	0.7	43.5	0.4	0.1	0.1	ND
**T19I** *	**C21618T**	0.1	2.5	0.1	30.8	0.1	98.6
**T19R** *	**C21618G**	ND	0.1	98.2	0.1	1.0	0.1
**DEL69/70**	**TACATG21765-21770DEL**	96.6	0.1	0.2	65.5	95.2	0.3
D80A	A21801C	ND	97.1	ND	ND	ND	ND
**T95I**	**C21846T**	0.3	0.1	38.0	64.9	95.0	0.1
G142D	G21987A	0.1	0.1	66.0	94.7	92.9	97.4
DEL144/144	TTA21994-21997DEL	95.0	0.7	0.2	0.1	0.1	0.1
DEL157/158	AGTTCA22039-22034DEL	ND	0.1	92.1	0.1	0.1	0.1
D215G	A22206G	0.1	94.6	0.1	0.1	ND	0.1
DEL241/243	CTTTACTTG22281-22289DEL	0.1	89.6	0.1	0.1	0.1	ND
**K417N**	**G22813T**	0.1	93.0	0.2	74.9	70.0	93.8
L452R	T22917G	0.1	0.1	96.8	0.2	0.2	0.1
**T478K**	**C22995A**	0.1	0.1	97.1	90.9	91.4	94.3
E484K	G23012A	0.3	86.4	0.1	ND	ND	ND
**E484A**	**A23013C**	ND	ND	0.1	90.7	91.4	94.3
**N501Y**	**A23063T**	97.6	86.9	0.1	88.2	89.9	91.0
A570D	C23271A	99.2	0.1	ND	ND	ND	ND
**D614G**	**A23403G**	99.3	97.7	99.3	99.1	99.3	99.5
**P681H** **	**C23604A**	99.0	0.1	0.1	98.7	99.0	99.2
**P681R** **	**C23604G**	0.2	0.1	99.1	0.1	0.1	0.1
A701V	C23664T	0.4	96.4	0.1	5.9	0.1	0.1
T716I	C23709T	98.7	0.2	0.1	0.1	0.1	0.1
D950N	G24410A	0.1	0.1	95.2	0.1	0.1	0.1
S982A	T24506G	98.7	0.1	ND	ND	ND	ND
**D1118H**	**G24914C**	99.2	0.1	ND	ND		0.1
N gene	C29208DEL	100.0	100.0	100.0	100.0	100.0	100.0

ND: Not detected, Bold: mutation site targets were used in both PMA panels

* These two mutation site used the same set of primer for amplification and extension.

** These two mutation site used the same set of primer for amplification and extension.

$ L18F is the hallmark mutation site for SARS-CoV-2 Gamma variant (97.9% prevalence)

# Data on 1 May 2022, outbreak.info

**Table 2:** Mutation prevalence across lineages of SARS-CoV-2 variant target sites used in PMA-Omicron panel.

Mutation site Amino acid	Mutation sites nucleotides	Mutation prevalence among lineage (%)^#^					
		Alpha	Beta	Delta	Omicron (B.1.529)	BA.1.1	BA.2
T19I*	**C21618T**	0.1	2.5	0.1	30.8	0.1	98.6
T19R*	**C21618G**	ND	0.1	98.2	0.1	1.0	0.1
A67V	C21762T	0.2	1.3	0.3	66.1	96.1	0.1
**DEL69/70**	**TACATG21765-21770DEL**	96.6	0.1	0.2	65.5	95.2	0.3
**T95I**	**C21846T**	0.3	0.1	38.0	64.9	95.0	0.1
DEL143/145	GTGTTTATT21987-21995DEL	ND	0.1	0.1	64.3	92.9	0.1
DEL212/212	ATT22194-22196DEL	ND	0.1	0.1	60.2	88.4	0.1
G339D	G22578A	0.1	0.1	0.1	93.8	93.1	96.1
R346K	G22599A	0.1	ND	0.1	32.5	92.1	0.1
S373P	T22679C	ND	0.1	0.1	89.9	88.6	95.8
S375F	C22686T	ND	ND	0.1	89.9	88.4	95.6
**K417N**	**G22813T**	0.1	93.0	0.2	74.9	70.0	93.8
S477N	G22992A	ND	0.1	0.1	90.8	91.3	94.3
**T478K**	**C22995A**	0.1	0.1	97.1	90.9	91.4	94.3
**E484A**	**A23013C**	ND	ND	0.1	90.7	91.4	94.3
Q493R	A23040G	0.1	ND	ND	90.2	92.1	92.3
Q498R	A23055G	ND	0.1	ND	87.7	89.4	90.8
**N501Y**	**A23063T**	97.6	86.9	0.1	88.2	89.9	91.0
Y505H	T23075C	0.1	ND	ND	88.1	89.5	90.8
T547K	C23202A	0.1	0.1	0.1	67.7	98.9	0.1
**D614G**	**A23403G**	99.3	97.7	99.3	99.1	99.3	99.5
H655Y	C23525T	0.1	0.1	0.1	99.0	99.2	98.5
P681H**	**C23604A**	99.0	0.1	0.1	98.7	99.0	99.2
P681R**	**C23604G**	0.2	0.1	99.1	0.1	0.1	0.1
D796Y	G23948T	0.1	0.1	0.1	97.7	97.7	98.9
N856K	024310A	ND	ND	0.1	67.4	98.7	0.1
Q954H	A24424T	ND	ND	0.1	98.4	98.3	99.4
N969K	T22469A	ND	ND	0.1	98.6	98.9	99.4
L981F	C24503T	ND	0.1	0.1	67.4	98.8	0.1
**D1118H**	**G24914C**	99.2	0.1	ND	ND		0.1
N gene	C29208DEL	100.0	100.0	100.0	100.0	100.0	100.0

ND: Not detected, Bold: mutation site targets were used in both PMA panels

# Data on 1 May 2022, outbreak.info

**Table 3:** Comparison of SARS-CoV-2 variant identification using Next Generation Sequencing (NGS) and MassARRY PMA-ABDO and PMA-Omicron (n = 358)

SARS-CoV-2 PCR Ct (N gene)	SARS-CoV-2 variant NGS examination (n)	PMA Panel assay	SARS-CoV-2 variant PMA Result (detected/tested)	
			MassARRAY call rate > 43.59%	MassARRAY call rate < 43.59%
**Group 1: SARS-CoV-2 positive sample with PCR Ct < 26 (n = 256)**				
< 26	Alpha (58)	ABDO	Alpha (58/58)	0/58
< 26	Beta (3)	ABDO	Beta (3/3)	0/3
< 26	Delta (112)	ABDO	Delta (112/112)	0/112
< 26	Gamma (1)	ABDO	Gamma (1/1)	0/1
< 26	Non-VOC* (23)	ABDO	Non-VOC (23/23)	0/23
< 26	Omicron (BA.1) (20)	Omicron	B.1.1.529 or BA.x (20/20)	0/20
< 26	Omicron (BA.1.1) (16)	Omicron	B.1.1 (16/16)	0/16
< 26	Omicron BA.2 (23)	Omicron	BA.2 (23/23)	0/23
**Group 2: SARS-CoV-2 positive sample with PCR Ct > 26 and NGS failed for variant characterization (n = 28)**				
28.33–35.54	Fail (14)	ABDO	Alpha (11/14)	possible Alpha (3/14)
29.33–39.94	Fail (9)	ABDO	Delta (9/9)	0/9
26.0–37.92	Fail (5)	Omicron	Omicron (B.1.1.529 or BA.x) (5/5)	0/5
**Group 3: SARS-CoV-2 positive sample with PCR Ct > 30 without NGS result (n = 74)**				
30.06–38.83	NA	ABDO	Alpha (41/41)	0/41
30–35.45	NA	ABDO	Delta (14/14)	0/14
30–39.1	NA	Omicron	Omicron (B.1.1.529 or BA.x) (19/19)	0/19

* List of non-VOC samples: One sample of B.1.1.1, B.1.1.10, B.1.158, B.1.275, B.1.466.2, B.1.5, B.1, B.40, B.6 and 14 of B.1.36.16

## Data Availability

The primer sequence data used in this study are available from Dr. Opass Putcharoen (opassid@gmail.com) but restrictions apply to the availability of these data, which were used under license (license application no. 2203001009) for the current study, and so are not publicly available. Data are however available from the authors upon reasonable request and with permission of Dr. Opass Putcharoen to any researcher wishing to use them for non-commercial purposes. Researchers who wish to obtain a copy of the data submit their request to opassid@gmail.com.

## Abbreviations

ABDO: Alpha, Beta, Delta and Omicron variants
CI: Confidence interval
COVID-19: Coronavirus disease of 2019
Ct: Cycle threshold
NGS: Next Generation Sequencing
PMA: Point mutation array
SARS-CoV-2: severe acute respiratory syndrome coronavirus 2
VDGs: Variant of concerns
WGS: Whole-genome sequencing
